# Third Annual Report of the Registrar-General of Births, Deaths, and Marriages in England, with Appendices

**Published:** 1842-04-01

**Authors:** 


### Medico-Chirurgical Review,

### No. LXXII.

**[No. 32 of a Decennial Series.]**

JANUARY 1, TO APRIL 1, 1842.

### THIRD ANNUAL REPORT OF THE REGISTRAR-GENERAL OF BIRTHS, DEATHS, AND MARRIAGES, IN ENGLAND, WITH APPENDICES. Presented to both Houses of Parliament by Command of Her Majesty. London, 1841.

THESE Reports are so calculated to do good service to medicine, that we have done and shall do our best for their dissemination. The body of facts that they contain must ultimately be of eminent utility. Nor is their application limited to a barren system of statistics, interesting to few and affecting the well-being of none. They bear upon the public health and contain the data for legislation in regard to it. They merit the close attention of medical men, not only as such, but as citizens.

The volume opens with a Report from the Registrar General himself to Lord Normanby, containing a general abstract of the Number of Births, Deaths, and Marriages registered during the year ending June 30, 1840.

The following statement will shew the numbers registered in the year ending June 30, 1840, compared with those of the preceding years:—

| | 1839-40. | 1838-39. | 1837-38. |
|---|---|---|---|
| Births . . . . | 501,589 | 480,540 | 399,712 |
| Deaths . . . . | 350,101 | 331,007 | 335,956 |
| Marriages . . . | 124,329 | 121,083 | 111,481 |

There is thus an increase in the number of Births registered in the year ending June 30, 1840, over those in 1838-9, of 21,049; over those in 1837-8, of 101,877:

In the number of Deaths registered in the year ending June 30, 1840, over those in 1838-9, of 19,094; over those in 1837-8, of 14,145:

In the number of Marriages registered in the year ending June 30, 1840, over those in 1838-9, of 3,246; over those in 1837-8, of 12,848.

The increase in the number of registered Births results from a continuance of that successful operation of the new law, which began to appear after the former half of the first year of registration.

In each of the last three years the proportion of Male and Female Births has been very nearly the same.

The Registrar General remarks:—

"The increase of deaths compared with those of the two preceding years is less than it appears to be. It must be remembered that the *first* year's registration could not comprise the deaths of the whole year, which, including those registered subsequently, amounted to 338,660. The real increase, therefore, over the registered deaths which *occurred in* the year 1837-8, is 11,441. It must also be borne in mind that the population of England and Wales, was shewn by the Censuses of 1821 and 1831, to have increased from 1821 to 1831 at the rate of 16 per cent.; and if it be assumed (as is probable) that this rate of increase has continued to the present time, the population in the years 1838-9 and 1839-40 will probably have increased to the amount of from 220,000 to 240,000 yearly. If the lowest of these numbers be taken, and the mortality be estimated at the lowest rate consistent with probability, namely, one in 50, there will in each of these years, at the same rate of mortality, have been at least 4,400 more deaths than in the year preceding. This number, therefore (being the probable increase, at the *same* rate of mortality) must be deducted; and the remaining numbers, which indicate *increased* mortality, will, for the year 1839-40 as compared with 1837-8, be about 7000; compared with 1838-9, about 14,700. Yet this increase is great; and inasmuch as there is reason to believe that it is not merely an *apparent* increase, arising, like that of births, from the improved efficiency of a registration which, at the commencement, was very defective (for the very efficient registration of deaths, even in the first year, left no such room for improvement), but that there has really been an increased mortality to that amount, a circumstance so serious demands attention and inquiry, with a view to ascertain the nature of the increase, and especially whether it has been sudden or progressive, general or local, and whether affecting equally or unequally all ages and both sexes." 4.

The proportion of male to female deaths in each of the three years has been nearly the same, as appears from the following numbers:—

| | Males. | Females. |
|---|---|---|
| Year ending June 30, 1838 . . . . | 170,965 | 164,991 |
| ,, ,, 1839 . . . . | 169,112 | 161,895 |
| ,, ,, 1840 . . . . | 177,929 | 172,172 |

The increase has been principally in the deaths of children, for more than half the excess over the deaths of 1838-9, and more than three-fourths of the excess over those of 1837-8, consisted of the deaths of children under 5 years of age.

This increase of mortality has been confined to certain situations, others exhibiting a decrease.

Thus there has been a progressive decrease of mortality from 1837-8, in the metropolis and in Devonshire; and a progressive increase from 1837-8, in divisions 15, 18, 19, 20, 21 and 25, comprising the counties of Derby, Leicester, Northampton, Nottingham, Rutland and the northern parts of Lincolnshire; the counties of Chester, Salop, and Stafford, except the mining parts of the two latter; Lancashire, south of Morecambe Bay, except Liverpool and Manchester; the West Riding of Yorkshire, except the northern parts thereof and Leeds; the City, Ainsty, and East Riding of York, Monmouthshire, Herefordshire, and Wales.

The counties in which there has been the greatest increase of mortality, compared with that of 1838-9, are Lancashire, Nottinghamshire, West Riding of Yorkshire, Leicestershire, Cheshire, Gloucestershire, Northumberland, Durham, Derbyshire, and North Wales, the combined increase of which alone amounts to 15,231, out of the total increase of 19,097.

The prevalence of such increased mortality in those counties which comprise the largest proportion of manufacturing population, naturally suggests that the cause may probably be found in circumstances to which the manufacturing classes have been peculiarly exposed. But a further examination shews that not only has the increase varied very much within those counties, but that there has even been a decreased mortality in some of those districts which are peculiarly the seats of manufacture. Such has been the case in Manchester and Salford, Ashton, Oldham, Stockport and Leeds. Therefore, though manufacturing distress cannot be excluded from among the possible causes of increased mortality, care must be taken not to assign such mortality to this one cause in an undue degree.

It appears that this increased mortality is attributable chiefly to the prevalence of epidemics, especially of typhus and scarlet fever, and that the districts of Chorley, Leigh, Wigan, Burnley and Blackburn in Lancashire; Macclesfield, Dewsbury, Pontefract, Nottingham, Bingham, Ashby-de-la-Zouch, Bangor, and Beaumaris, are those in which these diseases have been most fatal.

The number of marriages would seem to have augmented in some slight degree. The counties in which early marriages prevail are "Hertford, Bedford, Cambridge, Huntingdon, Northampton, Leicester, and Essex." The same, with the addition of Wiltshire, are the eight counties in which, in the succeeding year, are the largest proportion of men married under the age of 21. The least *matrimonial* counties are Hampshire, Devonshire, Herefordshire, Shropshire, East and North Riding of Yorkshire, Durham, Cumberland, and Westmoreland.

From the writing *test* on marriage, the counties at the minimum and maximum of the educational scale would appear to retain their respective positions—the Metropolis and Cumberland standing at the head, Bedford and Monmouth at the bottom of the list.

An analysis of 10,019 marriages makes the average age of marrying in men 27·4 years, and in women 25·5 years.

A Table shews the proportion of deaths at different ages out of 1,000 registered deaths for each division and for the whole of England and Wales.

The deaths of children (taking the mean of both sexes) under 1 year of age, which everywhere constitute so large a portion of the whole mortality, sometimes exceeding even a fourth, appear to have been comparatively most numerous in the mining districts of Staffordshire and Shropshire; the south of Lincolnshire, Huntingdonshire, and Cambridgeshire; the manufacturing parts of Lancashire and Yorkshire; in Manchester, Liverpool, and Leeds; in Norfolk and Suffolk; and in the division comprising the north of Lincolnshire, Rutland, Derbyshire, Nottinghamshire, Leicestershire, and Northamptonshire; in all which divisions the proportion has ranged from 238 to 293. In Birmingham the proportion has been a little less, and much less in the Metropolis. The smallest proportion of deaths under that age has been in Devonshire, Dorsetshire, Wiltshire, Bedfordshire, Lancashire north of Morcambe Bay, Westmoreland, Cumberland, Northumberland, and Durham (excepting the mining parts of each); the North Riding of Yorkshire; Herefordshire, Monmouthshire, and Wales; in all of which the number has ranged from 168 to 186.

These results have been most similar to those of the preceding year for the whole of England and Wales; for the Metropolis, Liverpool, Leeds, Dorsetshire, and Wiltshire; the district comprising the southern part of Lincolnshire, with Huntingdonshire and Cambridgeshire; the district comprising the remaining part of Lincolnshire, with the counties of Rutland, Nottingham, Derby, Leicester, and Northampton; the mining parts of Northumberland and Durham; the northern counties; and for Herefordshire, Monmouthshire, and Wales. The principal differences elicited by comparison have been in Birmingham, Devonshire, Cornwall, Norfolk, and Suffolk, Staffordshire, Shropshire (except the mining parts), and Cheshire and Lancashire (except Liverpool and Manchester) south of Morecambe Bay.

The proportion of deaths at advanced ages has been greatest in Devonshire, Dorsetshire, Wiltshire, Cornwall, in the counties north of Yorkshire, and in Norfolk and Suffolk. It has been least in Liverpool Manchester, Leeds, Birmingham, and the mining districts of Staffordshire, and Shropshire.

Such is the substance of the Report of the Registrar-General. The Tables on which it is founded follow, and these are succeeded by an Appendix on the causes of death in England and Wales, by Mr. Farr.

### ON THE CAUSES OF DEATH IN ENGLAND AND WALES.

The deaths in the year 1839, were not so numerous by 3,550 as those in 1838, but, in consequence of the better registration, the number of cases in which the causes were specified scarcely differed in the two years.

In 1838 the causes of 330,559 deaths were stated.
1839 „ 330,497 „

The mortality was lower than in 1838, the diminution being 2·4 per cent. among males, and 2·6 per cent. among females, or exactly $2\frac{1}{2}$ per cent. in the two sexes. The Winter of 1838 was extremely cold, the mean temperature of January being eight degrees, and that of February six degrees below that of the corresponding months in 1839.

The number of deaths from the class of *epidemic, endemic, and contagious diseases*, was 65,343; and the mean rate of mortality per 1,000 by the class was 4·25; in 1838 it was 4·52. The decrease was in small-pox and typhus, 16,268 persons having died of small-pox in 1838, and 9,131 in 1839; 18,775 of typhus in 1838, and 15,666 in 1839. On the other hand, 6,514 children died of measles, and 5,802 of scarlatina, in 1838; while 10,937 died of measles, and 10,325 of scarlatina, in 1839. Hooping-cough declined. Croup, thrush, diarrhœa, dysentery, cholera, influenza, and erysipelas remained stationary; none of them assumed the epidemic form. Ague rose from 44 to 95. In 1838, 16 males and 8 females died of hydrophobia; in 1839, 11 males and 4 females perished in the same way. Out of a population of 100,000 of each sex, 432 males and 418 females died of the epidemic class of diseases; but when the comparison is instituted between the deaths alone the proportions are reversed; in 100,000 deaths of males 19,368, and in 100,000 deaths of females, 20,189 were from the same class of diseases.

*Violent Deaths.*—On this head, Mr. Farr has collected a great deal of curious information.

It appears that 12,055 deaths were referred to "external causes or violence" in 1838, and 11,980 were classed under the same head in 1839. The number of suicides was less in 1839 than in 1838; but the proportions at different ages, in different parts of the country, and in the seasons of the year, remained unchanged.

The tendency to commit suicide appears to increase up to the age of 60, and to be then more than three times as great as at the age of 25.

Exclusive of 2,001 deaths by suicide, 22,034 deaths were referred to external causes in the two years 1838-9; and it will be recollected that many cases of lock-jaw, erysipelas, mortification, hernia, and other diseases, are the results of injuries and of accidental violence.

The mortality from violence ranges from 509 to 1,015 in 1,000,000, and it is highest in the mining and manufacturing, lowest in the agricultural districts. The metropolis occupies an intermediate place.

Out of a population of 1,000 males in *England and Wales*, 1·064 died a violent death in 1838; and 1·053 in 1839: or, without decimals, 1,064 in 1,000,000 males died violent deaths in 1838.

In the northern counties 16 in 10,000 *males* die violent deaths annually; in Staffordshire, Warwickshire, and the other western counties, 14; in Lancashire and Cheshire, 13; in Cornwall and the other south-western counties, 11; in the metropolis, 8; in Essex, Suffolk and Norfolk, 7. The deaths of *females* by violence are most numerous in Cheshire, Lancashire, the western counties, and Yorkshire, where they are employed in factories, and are exposed to accidents by machinery. In Kent, and the other south-eastern counties, 3 females in 10,000 died violent deaths; in Lancashire and Cheshire 5 in 10,000 annually.

The mortality of males from violent deaths was to that of females as 26 to 10; the tendency to suicide was as 23 to 10, or nearly in the same proportion.

Two-thirds of the males were aged 20 and upwards, while less than half of the females were of that age. Under 20 the number of males was 1,311, the females 853; at the age of 20 and upwards the males were 2,650, the females 705. Nearly half (5315) of the total violent deaths in the country happen to men above 20 years of age; 44 per cent. are 20 and under 60: so that, exclusive of suicide, and the deaths at sea, 4367 men in the prime of life are cut off every year in England by injuries and accidents of various kinds.

Of 162 ascertained suicides, of the age of 20 and upwards, whose occupations were stated, 18 were labourers, 10 tailors, 8 shoemakers, 6 seamen (1 of the 6 was a commodore, 2 captains), 5 licensed victuallers, 5 servants, 4 merchants, 4 coachmen, 4 bakers, 4 paupers, 3 medical men, 3 officers or soldiers, 3 clerks, 3 engravers, 3 cheesemongers, 3 weavers, 3 smiths, 3 masons, plasterers, or house painters, 3 gardeners, 2 attorneys, 2 watchmen, 2 beadles, 2 printers, 2 moulders, 2 saddlers, 2 tobacconists, 2 shopmen; and there was *one* of each of the following;—sculptor, artist, teacher of music, translator of languages, architect, bookseller, copper-plate printer, colourer of prints, book-keeper, corn-dealer, cattle-dealer, coal-dealer, farrier, coach proprietor (formerly), horse-keeper, cooper, carpenter, painter and glazier, cabinet-maker, coachmaker, coach-plater, chair-maker, cane-worker, pencil-maker, pianoforte-maker, gun-maker, pocketbook-maker, comb-maker, parchment-maker, boiler-maker, brass-founder, brass-finisher, gold refiner, jeweller, water-gilder, china-mender, dyer, grocer, green-grocer, currier, dresser of Spanish leather, hair-dresser, hemp-dresser, lodging-house keeper, licensed hawker, milk-man, porter, patrol, waiter, potboy, dealer in old clothes. The professions of 8 suicides were not stated; 4 were registered "gentlemen."

Of 508 men, aged 20 and upwards, who died by other violent deaths, or by homicide, and whose occupations were stated, 143 were labourers, 86 mariners, watermen, fishermen, or barge carters, 25 masons, builders, plasterers, house-painters, or slaters, 11 carpenters, 4 shipwrights, 3 sawyers, 4 painters, glaziers, and plumbers, 21 engineers, stokers, or firemen, 15 servants, 13 coachmen, cabmen, or postboys, 10 paupers, 7 shoemakers, 7 pensioners, 6 bakers, 6 weavers, 5 smiths, 5 tailors, 5 porters, 4 livery stable-keepers, 3 soldiers, 3 policemen or watchmen, 3 gardeners, 3 custom-house or excise officers, 3 coal-dealers and whippers, 3 excavators, 3 butchers, 3 musicians, 2 merchants, 2 stockbrokers, 2 clerks, 2 fishmongers, 2 farriers, 2 ostlers, 2 messengers, 2 mechanics, 2 cutlers, 2 coopers, 2 tide-waiters, 2 schoolmasters, 2 cordwainers. Of no other occupation was there more than one. The occupations of 32 were not specified; 7 were registered as "gentlemen."

Nearly 12 men in 10,000 (exclusive of sailors and soldiers), aged 20 years and upwards, died violent deaths in the metropolis; and from the best estimate that can be made from the verdicts recorded in the registers, 5 of the 12 were suicides. *One* in 2,000 men committed suicide in the year.

The tendency to suicide is least among persons who carry on occupations out of doors; and greatest among artisans who are weakly from birth, are confined in-doors, have their rest disturbed, or have little muscular exercise.

Taking the numbers as they stand, 1 in 9,332 masons, carpenters, and butchers committed suicide in the year; and 1 in 1,669 tailors, shoemakers, and bakers: the tendency to suicide in the first class was as 1·0 to 5·6 in the second. The corrected mortality from suicide was 1·33 to 10,000 in the first class, and 7·43 to 10,000 in the second class.

A similar result is obtained by comparing the suicides in the class of labourers with those among artisans and tradespeople; for the tendency to suicide is twice as great among artisans as it is among labourers.

The proportion of suicides in the miscellaneous class, designated by Mr. Rickman, "*capitalists, bankers, professional, and other educated men,*" is very near the average.

Mr. Farr adverts to the opinion that the increase of education leads to an increase of suicide. He observes:—

"In England suicide is, in fact, most frequent in the metropolis, the south-eastern counties, and the northern counties, where the greatest number can write; and it is the least frequent in Wales. The intermediate counties range from 62 to 48, who could write, in 100; the suicides, from 4·5 to 6·8 in 100,000.

There is a general but no constant relation between the state of education thus tested, and the commission of suicide. It may be admitted that there is some relation between the development of the intellect and self-destruction; but the connexion must be in a great measure indirect and accidental. In opposition to the arguments derived from agricultural districts, and labourers in towns, there is the fact that suicide is more frequent among several classes of artisans, than it is among better educated people. If the progress of civilization is to be charged with the increase of suicide, we must therefore understand by it the increase of tailors, shoemakers, the small trades, the mechanical occupations, and the incidental evils to which they are exposed, rather than the advancement of truth, science, literature, and the fine arts."

This opinion of Mr. Farr's has met with criticism, we think, rather unfairly. His facts, so far as they go, prove his case. If suicide be not most rife in the educated classes, it is surely unjust to lay the blame of it on education. That the modern social system gives birth to it may be true, but probably that is on other grounds than on those of mental cultivation. It is to the inequality of conditions in life, to the near and painful contrast of poverty and riches, to vice and to intemperance, with their calamitous consequences to the moral nature and physical well-being of men, that we must look for the prevalence of suicide. And when, in wealthy countries like our own, there shall be no squalid misery, nor sudden descents from affluence to want, when the streets shall not be crowded with the victims of seduction, and the gin-palaces shall be empty of the votaries of excess, when improvidence shall cease to ruin, and penury shall be comfort, then, and not before, whatever be the education of the country, suicide will be but rarely seen.

Mr. Farr goes on to remark:—

"Intemperance and suicide, as well as other violent deaths, are found associated in the registers; and the professions peculiarly addicted to drunkenness have more than the due proportion of suicides. Drunkenness leads to this; but drunkenness is a sort of indirect suicide, and both are tendencies of the mind, indulged often from the same motives, and promoted by similar causes; for in drunkenness the wretched find not only the gratification of an appetite, but the suspension of natural consciousness—in death they seek its cessation.

There is no reason to believe that suicide has been latterly increasing in England. The fact, nevertheless, that 1000 persons are ascertained to commit suicide yearly, and that nearly as many more are returned as drowned, &c., in which the verdicts do not state whether death was accidental or suicidal, is sufficient to arrest attention on all the relations of the question.

Some plan for discontinuing, by common consent, the detailed, dramatic tales of suicide, murder, and bloodshed in the newspapers is well worthy the attention of their editors. No fact is better established in science than that suicide (and murder may perhaps be added) is often committed from imitation. A single paragraph may suggest suicide to twenty persons; some particular, chance, but apt expression, seizes the imagination, and the disposition to repeat the act, in a moment of morbid excitement, proves irresistible. Do the advantages of publicity counterbalance the evils attendant on one such death? Why should cases of suicide be recorded at length in the public papers, any more than cases of fever? It would be out of place to refer here to the moral or strictly medical treatment; but it may be remarked, that the artisans most prone to suicide are subject to peculiar visceral congestions—that suicide is most common in unhealthy towns—and that the influence of medicine on the mind, and on the unstable ungovernable impulses which are often the harbingers of suicide—is incontestable. To place the shoemaker, tailor, baker, or printer in the same favourable circumstances with respect to air and exercise as carpenters and masons would be impossible. But the workshops of all artisans admit of immense improvements in ventilation. Cleanliness is greatly neglected. Neither the men nor all the masters appear to be aware that the respiration of pure air is indispensable; that the body requires as much especial care as the tools, instruments, and machines; and that without it, neither the body nor the mind can be preserved in health and vigour. The new parks and public walks will afford the artisan an opportunity of refreshing his exhausted limbs and respiring in the fresh air; and the health and temper of the sedentary workman may be much ameliorated by affording facilities in towns for athletic exercises and simple games out of doors, which, while they bring the muscles into play, unbend, excite, and exhilarate the mind. Moral causes, and the regulation of the mind, have perhaps more influence on the educated classes; but all must derive benefit from out-door exercise."

The mortality of males, aged 20, from violent deaths, exclusively of suicide, was 6·77. As a general rule the suicides were most numerous in the trades least exposed to accidents; as if the mind, left unexcited by natural dangers, imagined and created causes of death. Three in 10,000 tailors, bakers, shoe-makers, and 9 in 10,000 masons, carpenters and butchers, were killed by accidents or violence—the reverse of the proportions in suicide. The degrees of danger which beset the different classes of the community in their occupations, are shewn by the following facts:

86 sailors, watermen, or fishermen, died violent deaths. The occupation of engineer is at present the most dangerous followed; 21 engineers, stokers, and firemen, were killed in one year in the metropolis, and that chiefly in the steam-vessels on the Thames.

Mr. Farr observes, that violent deaths may be considered under three points of view:—1st, With relation to the injury, which is the proximate cause of death; 2nd, The agents by which the injury is inflicted; 3rd, The circumstances which led to the death.

About 2454 persons are drowned every year in England and Wales, and it is computed that, in addition, about 1000 lives are annually lost by shipwreck, in sailing vessels only. The following Table is not devoid of interest.

*Table of the Violent Deaths in the Metropolis* (1838-9), *Manchester, Salford, Liverpool, West Derby, Birmingham, Norfolk, Suffolk, and Mining Districts.*

| | | |
|---|---|---|
| Mechanical Injury. | Gunshot Wounds | 47 |
| | Other Wounds with loss of blood | 132 |
| | Fractures and Contusions | 1,644 |
| | Total | 1,823 |

| | | |
|---|---|---|
| Chemical Injury. | Lightning | 5 |
| | Explosions | 42 |
| | Burning | 962 |
| | Scalding | 201 |
| | Poisoning, by Opium | 31 |
| | ,, Arsenic | 17 |
| | ,, Medicines improperly given ,, Other substances, & those not stated | 134 |
| | Total | 1,392 |
| Suspension of Respiration (Asphyxia.) | Drowning | 1,044 |
| | Hanging and Strangling | 253 |
| | Inhaling Mephitic Gases | 15 |
| | Suffocation | 144 |
| | Total | 1,456 |
| | Other Causes, and not specified | 297 |
| | Grand Total | 4,968 |

The violent deaths of men whose occupations are carried on above the level of the earth are most generally the effect of falls. Thus, in the metropolis, 15 of 19 violent deaths among masons, plasterers, and slaters; 6 in 11 carpenters; 2 in 2 deaths of painters and glaziers, were caused by falls; also 9 in 14 servants. Of 8 violent deaths among coachmen and postboys, 6 were from falls. Of 100 labourers, 22 died by falls, 18 by drowning, 12 by being run over, and 22 by being crushed. Miners and persons who work much below the level of the earth, are liable to be killed by the fall of heavy substances; 118 in 870 violent deaths which occurred in the mining districts, were caused in this way. Miners are sometimes killed by falling into pits; 12 were killed in a coal-pit at Clutton, the rope having been intentionally cut. Of 47 violent deaths among sailors and watermen, 7 were by falls, and 33 by drowning; the latter being of course almost always the result of *falls* into the water. Burns, from their clothes taking fire, are the most common causes of violent deaths in females; 77 males and 159 females (two-fifths of the females who died by violence), died of burns in the metropolis.

*Drowning*.—This is probably the most common mode of death in suicide; but the inquests in the metropolis left it undetermined whether the drowning was voluntary in 191 cases. 21 males, and 8 females, were stated to have committed suicide by drowning; 67 males, and 24 females, by hanging; 28 males, and 17 females, by poisoning; 6 males, and 4 females, by throwing themselves from windows and heights; 42 males, and 5 females, by wounds; 10 males, and no females, by gun-shot wounds. Of 3,146 cases of violent death, 2,371 were pronounced accidental; 388 were ascribed to human agency; and, in 387 cases, the verdicts did not state whether the death was the result of suicide, accident, or murder.

*Are Violent Deaths on the Increase?*—Something like an approximation to an answer may be got from the London Bills of Mortality from the middle of the 17th century.

In the first period (1647—1700) the annual rate of mortality was about 7, in the second 5·2, in the third 5, in the fourth 3, per cent.; whence it may be deduced that, in the 17th century 6·8 in 100,000, in the 18th century 5·4, in the 19th century 5, died violent deaths. Out of a given amount of population the deaths by drowning increased in the latter half of the 18th century; the deaths by scalds and burns were twice as great in 1800—1830 as in the 17th century. The tendency to suicide remained nearly stationary; so did death by poisoning. All the deaths by personal violence rapidly decreased. In a population of 100,000, according to these accounts, about 23 were killed, 4·6 murdered, in the 17th century; in the 19th century about 13 were "killed," and 0·5 were murdered. The chance of being murdered diminished nine-fold. The executions were more frequent in the latter half than in the beginning of the 18th century; compared with the population within the Bills of Mortality, they were not however half so frequent in the first 30 years of the 19th century as in the latter half of the 18th century, when about 7 were executed annually to a population of 10,000. Relatively to the murders the number of executions increased.

From the Swedish, Prussian, and French returns, it appears that the mortality by violent deaths is greater in England, where it exceeds that of any country not devastated by civil war. The following suggestions of Mr. Farr deserve attentive consideration.

"Deaths in ships, manufactures, and mines, are indiscriminately called 'accidental;' yet it is well known that fewer lives are lost by shipwreck in Her Majesty's service than in emigrant vessels; that less accidents happen in one factory than in another; and that the men are crushed, burnt, or blown to pieces, much less frequently in the coal-mines of certain proprietors, than in those of others. Many 'accidental deaths' are, therefore, *indirectly caused* by human agency. Many of the accidents happen from ignorance and carelessness. The knowledge of the accidents to which people are exposed in different occupations may put them more on their guard against danger. Men who work at a considerable elevation from the earth will learn caution from the number of deaths by falls, and will, perhaps, indulge less in intoxicating drinks (which are the cause of so many accidents), or in anything that makes the step or head unsteady. In the metropolis, in two years, 142 males and 285 females, died by burns! This is to be ascribed to the greater combustibility of the dresses of females: their caps and gowns frequently take fire. Many children are burnt from the same cause. It deserves the consideration of manufacturers, whether cotton and linen may not be made, by a chemical solution, as little liable to take fire as textures of wool. It may render parents and servants more careful to state that many children, under five years of age, are suffocated by drinking boiling water out of the tea-kettle—are burnt to death—or disfigured for life—from being left alone at the fire, without a guard; and that many children are poisoned by drinking medicines, or drugs, left within their reach. 500 or 600 persons are ascertained to die by poison every year in England; besides the cases of poisoning which are never detected. These are not like the other violent deaths. The poisons are of very little use except in the hands of medical men; and may, without any disadvantage, be placed beyond the reach of the majority of persons by whom they are employed for self-destruction, or murder. Arsenic, mixed with food, cannot be tasted, and is fatal in very small quantities; yet it is obtained with almost as much facility as sugar, by servant-girls, in the small chemists' shops. About 100 fatal cases of poisoning, by arsenic, are detected every year. It is generally asked for 'to kill rats;' but it is questionable whether arsenic kills more rats than human beings: and, if the destruction of rats is a matter of so much importance, it may be effected in other ways. The suicide, or murderer, would, it is true, often resort to other means, if poison were inaccessible; but he would not always do so; and many of the 'accidental deaths,' which now occur by taking poison by mistake, would be prevented. The taste of opium cannot easily be disguised: hence it is less used by the murderer than the suicide. Small quantities of opium are fatal to infants: and mothers and nurses, frequently give children over-doses of laudanum, or elixir, and quack medicines, in which it is mixed up in uncertain quantities. It is admitted by those who have paid most attention to this subject, that the system of pharmacy in England, and the sale of poisons, requires revision. The sale of prussic acid, opium, nux vomica, oxalic acid, corrosive sublimate, and arsenic, to the public, may be prohibited, or be permitted only by medical prescription. The master's certificate may be required for sugar of lead, and poisonous substances employed in the arts and manufactures. The immense number of deaths by drowning (about 2,400 annually), arises, in part, from the neglect of the art of swimming, even by persons who are frequently on deep waters.

The fatal effects (either direct or remote) of inhaling different kinds of mephitic gases, such as sulphuretted hydrogen, carburetted hydrogen, carbonic acid, and nitrogen, given off from cess-pools, lime-kilns, brick-kilns, crowded assemblies in ill-ventilated rooms, and graves, are tolerably well known; nevertheless, violent deaths still occur from them in the metropolis. A grave-digger and a fishmonger it has been already stated perished in one of the London churchyards. The carburetted hydrogen and carbonic acid are rarely dense enough to prove immediately fatal in coal mines; but in small doses they are slow poisons, —they give rise to diseases: and admitting fully the power of Davy's admirable invention, the safety lamp, to enable miners to breathe carburetted hydrogen without producing explosion, no choice can exist between the employment of this lamp, and an efficient system of mechanical ventilation, by which all the insalubrious gases may be removed, and the men may be supplied in the remotest parts of the mines with pure air."

In this country, where human life is estimated at its maximum, and where humanity characterises public opinion so strongly, it is only requisite to state the facts collected by Mr. Farr, in order to awake attention to them. Unquestionably many "accidents" are negligence, and culpable negligence on the part of employers or public bodies. This is a case for the interference of the legislature, which has held back too much in such matters in this country.

### *Murders.*

The murders which have been registered and noted in the abstracts, on the authority of the verdicts of coroners' juries, amounted in two years to 156 (males 103, females 53.) The proportion to the population is 5 in a million annually, or 1 in 200,000. Of 148 persons murdered, whose ages were abstracted, 78 were aged 20 and upwards. If we assume that half of the population is above 20 years of age, about 5·3 in a million adults were murdered annually. The number of cases in which verdicts of murder were brought in, was above the average in the south-western counties, in Lancashire and Cheshire, the south-eastern counties, the north midland counties, and the metropolis. If infants be excluded, the greatest proportion of murders occurred in Lancashire and Cheshire, the smallest proportion in Essex, Suffolk, and Norfolk.

### *Lightning.*

25 persons (18 males, and 7 females) were killed by lightning in 1838, and 18 (14 males, and 4 females) in 1839. In the last two quarters of 1837, *fifteen*; of 1838, *fourteen;* and of 1839, only *two* deaths were caused by lightning. In the two years (1838-9) 1 of the deaths occurred in May, 26 in June, 8 in July, 4 in August, 2 in September, and 2 in November. *Six* was the greatest number killed in one storm, which happened on the 18th of June, 1839; and it is a curious coincidence that *four* persons were struck dead by lightning on the 18th June, 1838.

The danger of being struck by lightning is comparatively not very great; for only 22 are killed by this cause in the year, while 29 die other violent deaths *daily*, exclusive of suicides, Of a million men at the age when they are most exposed (30—50), not more than 4 were struck dead; while the proportion of women was less (1·5). During the thunderstorm, however, the danger of death by lightning is probably twice as great as the danger at that time from all the other causes of violent death put together. Franklin wrote popular directions as to the best means of protection in storms, but it appears from M. Arago's interesting paper, that natural philosophers are not agreed altogether in admitting their propriety. Unless there is something in the structure or dress of men which marks them out in a special manner as victims of lightning, it may be inferred from the facts in the registers that people are safest in-doors when it lightens; and that women and children are placed in the circumstances where there is least danger.

### *Sudden Death.*

Mr. Farr considers, under this head, cases of sudden death in which inquests were held, but the cause of death was not ascertained. Sudden deaths of this class are more frequent among males than females. The ratio is nearly as 16 to 10. About 16 happen in Winter to 10 in Summer. Mr. Farr gives an analysis of 424 verdicts. The cause of nearly 2 in 3 sudden deaths is not stated in the verdicts. Apoplexy was said to be the cause of 53 deaths, diseases of the heart and arteries of 27, exclusive of 10 ascribed to rupture of a blood-vessel. Fits and convulsions come next in the order of frequency. Several of the sudden deaths happened in the course of chronic diseases, but the cases of consumption occurred principally among criminals in the prisons, and were not sudden deaths. In 1087 other cases which occurred in the metropolis (1839), the deaths were ascribed to apoplexy 84 times, convulsions 17 times, epilepsy 10 times, heart diseases 36 times, and rupture of a blood-vessel 36 times. The verdicts, "visitation of God," or "natural death," were returned 632 times in the 1087 inquests, not comprising violent deaths.

Mr. Farr inquires "what are sudden deaths?" and does not seem able to define them very satisfactorily. Certain it is that they are not so commonly apoplexy as is imagined.

M. Devergie, who is Medical Director of the *Morgue,* to which all bodies found dead in Paris are conveyed, and who has had better opportunities for investigating the subject than any other person, refers sudden death, after Bichat, to the three principal organs—the lungs, brain, and heart. In death by the lungs, the circulation is stopped primarily in those organs; the pulmonary artery, the right cavities of the heart, and the *venæ cavæ* are gorged with blood. The pulmonary veins, the left cavities of the heart, and the aorta are empty, or contain an infinitely small portion of blood. In death by the brain (apoplexy), the respiration is embarrassed, the lungs congested, and then the heart ceases to beat; the meningeal veins are gorged with blood; the lungs contain a considerable quantity; there is blood in both sides of the heart, but most in the right cavities. If death begin at the heart (*syncope,* fainting), its action ceasing all at once, the cavities are full on both sides, not as they are in the state of accumulation, but as in the ordinary state of the circulation; there is blood in the arteries and veins. Neither the lungs nor the brain are congested. This is a brief summary of the results of M. Devergie's researches. The term congestion is vague, and by no means unobjectionable; but it is often all that is found in violent death by asphyxia, and it has something like a specific meaning in M. Devergie's essay; who reports several cases at length, which medical witnesses will do well to consult.

The following is a summary of 40 cases of death, carefully examined by the Medical Director of the *Morgue:*—

Apoplexy, with a clot in the annular protuberance, 1; meningeal apoplexy, 3; serous apoplexy and pulmonary congestion, 2; congestion of the brain and spinal marrow, 3; pulmonary congestion, 12; pulmonary and cerebral congestion, 12; hæmatemesis, 2; syncope, 3; rupture of the heart, 1; rupture of the pulmonary artery, 1.

Mr. Farr inquires into the proper business of a Coroner's Jury. It is provided in the Registration Act that "in EVERY CASE *in which an inquest shall be held* on any dead body, the *Jury shall* INQUIRE OF THE PARTICULARS HEREIN REQUIRED TO BE REGISTERED *concerning the death,* and the coroner shall inform the Registrar of the finding of the jury, and the Registrar shall make the entry accordingly." One of the particulars required to be registered is "the CAUSE of DEATH." Now this cannot be certainly determined without an examination of the body, and it is to be hoped that this will be more generally insisted on by Coroners than it has been. Mr. Farr observes:—

"It would be taking a narrow view to assume that the inquest is intended only to *detect* deaths by murder. About 35,000 inquests were held in the two years, 1838-9; when 156 murders were registered. There was but one verdict of murder to 224 inquests. According to the Criminal Returns, 121 offenders were tried for murder in 1838-9; of that number only 37 were convicted, and 15 were executed. The number of deaths by manslaughter is inconsiderable. The primary question in every inquest unquestionably is:—Was the death the result of homicide? And even this can only be satisfactorily answered by strictly complying with the provision of the Registration Act, and inquiring into the particulars of the actual cause of death. Exclusive of the use of the deodand in preventing accidents, however, the principal utility of the inquest is the security which it affords the public mind; and its tendency to prevent crime, by convincing the evil-minded that murder cannot be committed with any chance of impunity. But inquests, in which the 'cause of death' is not inquired into, can neither inspire criminals with dread, nor the public with confidence. The most important part of the evidence of the inquest is omitted, when the 'cause of death' is not investigated. The expense of inquests, which is now not considerable, would be slightly augmented; but the value of the information, and the use of the inquiry, would be increased in an infinitely greater degree. The Legislature, moreover, has left the coroners no discretion upon this matter. The juries are bound by the Act to inquire into the particulars required by you; and the coroners are bound to supply the registrars with the results—comprising an intelligible statement of the 'cause of death,' so far as it can be ascertained. The inquests in England will, henceforward, be as efficient as similar inquiries in France or Germany; and be placed on a level with the present state of medical jurisprudence,—to contribute to that branch of science an immense number of new, well-authenticated, and instructive facts."

Nothing can be more just than this.

Mr. Farr dwells on the facility which drowning affords the purposes of the murderer. The individual is "found drowned," and that is all that can be said. The body is conveyed to some obscure pot-house for the jury to sit on it, and it is neither advertised nor very likely to be recognised. Mr. Farr recommends that some steps should be taken to remedy the defects in this part of our system of police. The bodies "found dead" should be conveyed to *one central place,* where they may lie until notice had been given to the coroners, and opportunities had been afforded for identifying them. Advertisements, drawn up under the direction of the coroners, should be sent to the newspapers, and placards to the police offices, before the inquests were held; which would enable the parties concerned to identify the dead, and the juries to ascertain the causes of death, in compliance with the clause in the Registration Act.

*Deaths in the London Hospitals.*

Abstracts have been made of the causes of death in the London hospitals, namely—in the Charing Cross, St. George's, Middlesex, North London, Westminster, St. Bartholomew's, London, Guy's, St. Thomas's, Greenwich, the Dreadnought, the Fever Hospital, and the Small Pox Hospital. The number of persons who died in those hospitals within the year amounted to 2491, of whom 1729 were males, 762 females. Of 301 persons who died of erysipelas, 60 died in the hospitals. In many cases the erysipelas must have followed operations or accidents; and it will be observed that 390 of 1212 deaths by violence were registered in these institutions. 7 of 20 deaths from aneurism, 31 of 73 from hernia, 7 of 15 from diabetes, 10 of 27 from stone, 41 of 123 from diseases of the joints, &c., 7 of 21 from fistula, 4 of 7 from purpura, 51 of 368 from carcinoma, took place in the hospitals. Hospital relief is not obtained equally at all ages. Sick children remain at home under the care of their mothers; they would in general be refused admission at hospitals. The aged poor, with chronic maladies, go to the workhouses. Young persons without families, between the age of 15—30, often servants and immigrants from the country, resort in the greatest numbers to the hospitals, where 1 in 7 deaths at that age are registered.

*Diseases of Towns and of the Open Country.*

Mr. Farr gives an unfavourable view of the health of Towns.

Thus the density of the country districts was to that of the towns as 10 to 245, the mortality as 100 to 144. The mean duration of life, in the two classes of districts, differs nearly 17 years; it is in the proportion of 55 years (country), to 38 years (towns). The difference is greater than was given in the calculation founded on the facts observed in 1838; when the deaths in Bristol, Clifton, and Norwich were (by error) not subtracted from the deaths in the counties of Gloucestershire and Norfolk. The mortality in the town districts, however, declined in 1839, more than the mortality in the country districts. As the population increases faster in the town than in the country districts, the difference in the mortality was greater than it is represented to be by these numbers.

The diseases, chiefly incidental to childhood, are twice as fatal in the town districts as they are in the country.

The deaths, by several diseases of old age, were almost equally numerous in the towns and the country. Asthma is, however, an exception.

The tendency to consumption was increased 24 per cent.—to typhus 55 per cent. in the town districts; but as the absolute mortality from consumption is three times as great as from typhus in towns, and nearly four times (3·73) as great in the country, the excess of deaths by consumption, caused by the insalubrity of towns, is greater than the excess of deaths by typhus—a fact which has hitherto been overlooked. Thus, 24,094 deaths from consumption occurred in the country, 32,436 in the town districts; the excess amonnted to 8,342 deaths; 6,402 deaths from typhus occurred in the country, 10,852 in the town districts; the excess amounted to 4,450 deaths. The difference is more correctly exhibited by a comparison of the mortality from the two diseases.

The facts show the propriety of the ordinary medical advice to place persons of a consumptive habit in a pure atmosphere; but they militate against sending them to reside in the continental towns, in many of which the mortality is as high as it is in Bethnal Green and Whitechapel. Paramenia (mismenstruation), though rarely fatal, is a very common disease, and one which greatly embarrasses the medical practitioner. The facts in the table point out the utility of the country watering-places to patients afflicted with the complaint in cities. The excess of deaths by childbirth in the town districts is striking. Out of nearly the same number of deliveries, 909 mothers died in the country, 1,560 in the town districts.

We are glad to perceive that the mortality in the whole of the metropolis—but particularly in Whitechapel and the worst parts,—had decreased in 1839. The Weekly Tables, up to the middle of 1841, show also a progressive amelioration; which may be expected, *cæteris paribus*, to proceed much more rapidly, when the new streets and park shall be opened, and when the sewers, and streets, and the dwellings of the poor are placed in circumstances which will secure more cleanliness and a moderate supply of pure air. The causes of the excessive mortality of towns are well known, and it cannot be too frequently repeated, that they admit of removal to a great extent.

Mr. Farr goes on to remark that, the mortality increases, *cæteris paribus*, as the density of the effluvial poison generated in cities, and not strictly as the density of the population. The indigence of the inhabitants, or an insufficiency of proper food—even when not carried to the extent of starvation or famine—has also a decided effect on the production of effluvial poisons, as well as on the tendency to diseases of every kind. Hence the mortality is not always greatest in the densest parts of cities.

This principle explains the facts that, although the mortality is increased 44 per cent. by the present condition of the towns in England,—where the proportion of town population is greater than in any other country in Europe, except Belgium,—the mortality of the nation has been much below the average during the whole of the present century; and, up to the present day, the expectation of life remains higher in England than in the rest of Europe. The industry and intelligence that have created flourishing towns have ameliorated, though not so rapidly as they might have done, the sanitary condition of the people.

*Diseases of different Parts of the Country.*

The mean mortality differs in several respects from that which obtained in 1838; when the metropolis, with its dense population (27,000 to the square mile), stood first in the table, the mean annual mortality having been 2·800, while that of Lancashire and Cheshire (with a population of 700 to the square mile) was 2·567. In the year 1839, the mean mortaliy of the metropolis fell to 2·371 per cent., and that of Lancashire and Cheshire rose to 2·836.

The high mortality among the population of Lancashire and Cheshire, from diseases of the nervous, respiratory, and digestive organs, observed in 1838, was sustained in 1839; and the increase in the mortality was occasioned by measles, scarlatina, and hooping-cough; for 383 in 100,000 died of these diseases in 1839, and only 133 in 100,000 in 1838. The difference is 250, which will make 2,817 when added to 2,567,—the mortality in 1838. In the metropolis the diseases of the nervous system and the respiratory organs were less fatal; but the greatest diminution occurred in small-pox and typhus. Yorkshire, and the division which comprises Nottinghamshire and Derbyshire, underwent a change analogous to that noticed in Lancashire and Cheshire. The mortality declined in the other divisions; and, as has been before stated, the mortality of the kingdom was lower in 1839 than in 1838.

*Influence of the Seasons.*

The disturbing circumstances of privations, epidemics, &c. operated as little as can be conceived possible on the inhabitants of the metropolis, during the three and a half years ending in June, 1841.

It is found that the degree down to which the mean monthly temperature falls in December, January, or February, determines, to a great extent, the mortality of Winter.

The January of 1838 was the coldest month of the 3½ years: the mean temperature of the two cold months—January and February—was nearly the same in 1839-40; and the Winter of 1841 was anticipated by the cold December of 1840, when the mean monthly temperature attained the minimum, and rose slowly through January and February.

The deaths registered in the seasons of the three years are compared below with the temperature expressed in degrees of the Fahrenheit and Centigrade thermometers. The number of deaths has been corrected, on the assumption that each period embraced 275 days.

| | 3 Winters. | 3 Springs. | 3 Summers. | 3 Autumns. |
|---|---|---|---|---|
| | January, February, March. | April, May, June. | July, August, September. | October, November, December. |
| Deaths .. .. .. | 39,764 | 35,128 | 33,677 | 36,684 |
| | ° | ° | ° | ° |
| Temperature (Fahrenheit).. | 39.8 | 53.8 | 61.0 | 44.6 |
| „ (Centigrade).. | 4.7 | 12.1 | 16.1 | 7.0 |

The causes of death which proved the most fatal in the cold months belong principally to the pulmonary class, and the cerebral diseases of the aged;—those which proved most fatal in Summer belong to the diseases of the bowels; but in almost every class there were one or two diseases over the fatality of which temperature exercised a marked influence.

Of the diseases in the epidemic class, influenza and hooping-cough followed the same law as the pulmonary; cholera, dysentery, diarrhœa, and thrush,—as the abdominal affections.

Persons affected by the following diseases died in greatest numbers when the temperature was low. It has been already rendered probable that many cases arranged under apoplexy and sudden death, are the effects of congestions in the lungs—a sort of spontaneous asphyxia, the development of which appears to be favoured by a temperature below the freezing point of water.

| Causes of Death. | Winter. | Spring. | Summer. | Autumn. |
|---|---|---|---|---|
| Apoplexy .. .. .. | 801 | 627 | 626 | 695 |
| Sudden Death .. .. | 618 | 524 | 381 | 547 |
| Paralysis .. .. .. | 647 | 520 | 485 | 602 |
| Insanity .. .. .. | 73 | 45 | 35 | 42 |
| Tetanus .. .. .. | 23 | 11 | 15 | 19 |
| Asthma .. .. .. | 1733 | 642 | 344 | 1080 |
| Bronchitis .. .. .. | 495 | 307 | 191 | 347 |
| Pneumonia .. .. .. | 3326 | 2454 | 1827 | 3600 |
| Pleurisy .. .. .. | 70 | 62 | 39 | 51 |
| Hydrothorax .. .. .. | 272 | 183 | 136 | 206 |
| Disease of Heart .. .. | 739 | 556 | 571 | 698 |
| Rheumatism .. .. .. | 124 | 113 | 99 | 117 |
| Nephritis .. .. .. | 19 | 17 | 14 | 19 |
| Diabetes .. .. .. | 19 | 12 | 7 | 15 |
| Dropsy .. .. .. | 1403 | 1286 | 1135 | 1457 |
| Diseases of Child-bed .. | 310 | 261 | 217 | 309 |
| Phlegmon .. .. . | 9 | 2 | 3 | 1 |
| Ulcer .. .. .. .. | 23 | 16 | 9 | 13 |
| Mortification .. .. .. | 217 | 177 | 153 | 171 |
| Old Age .. .. .. | 3437 | 2609 | 2150 | 2814 |
| Starvation .. .. .. | 21 | 16 | 7 | 20 |
| Violent Deaths .. .. | 996 | 989 | 883 | 924 |

The range of temperature in this climate appears to have little effect upon some of the fatal diseases of infants and adults.

| Causes of Death. | Winter. | Spring. | Summer. | Autumn. |
|---|---|---|---|---|
| Hydrocephalus .. .. | 1370 | 1330 | 1348 | 1231 |
| Convulsions .. .. .. | 2414 | 2298 | 2532 | 2119 |
| Consumption .. .. .. | 5600 | 5778 | 5501 | 5148 |
| Scrofula .. .. .. | 72 | 64 | 72 | 54 |
| Cancer.. .. .. .. | 276 | 230 | 264 | 262 |

As the corresponding seasons of different years present fluctuations in the temperature, this should, and a table proves that it does, supply further evidence of its influence.

The Autumn of 1840 was much colder than the Autumns of the two preceding years; the mortality of the diseases under the influence of temperature was raised in an equal degree.

| | AUTUMN. | | |
|---|---|---|---|
| | 1838 | 1839 | 1840 |
| | ° | ° | ° |
| Temperature { Mean . . | 45.2 | 45·6 | 42·9 |
| Temperature { Lowest. . | 29.8 | 32·0 | 21·2 |
| Deaths from Diseases of the Respiratory Organs, exclusive of Consumption } . . | 1730 | 1769 | 2361 |
| ,, Consumption . . . . . . . | 1729 | 1682 | 1737 |

"At what degree of cold," asks Mr. Farr, "does the mortality begin to rise? And how soon after the cold weather has set in is its effect experienced? The Weekly Tables of Mortality furnish replies to these questions. It will be recollected that the tables of the week comprise the *registered* deaths, about half of which must have *occurred,* when the rate of mortality was uniform in the week preceding.

Meteorologists have observed that the mean temperature of October represents very nearly the mean temperature of the year and the place; and the facts in the table show that the mortality rises progressively, as the mean temperature falls below the *mean temperature* of London (50°·5); the deaths in the week rising to 1000 and upwards when the *temperature* of night falls below the freezing point of water, and to 1200 when the *mean temperature* of day and night descends a degree or two lower than 32°.

The rise in the mortality is immediate; but the effects of the low temperature go on accumulating, and continue to be felt 30 or 40 days after the extremities of the cold have passed away. The cold destroys a certain number of persons rapidly, and in others occasions diseases which prove fatal in a month or six weeks. The relation of the temperature and mortality is distinctly shown in the next Table: where accidental irregularities are diminished by extending the number and the period of the observations. The practical lesson taught by these facts is obvious. A great number of the aged, and those afflicted with difficulty of breathing, whether it arises from emphysema, chronic bronchitis, diseased heart, or impairment of the function of respiration, cannot resist cold sunk so low as 32°. The temperature of the atmosphere in which they sleep can never safely descend lower than 40°; for, if the cold that freezes water in their chamber do not freeze their blood, it impedes respiration, and life ceases when the blood-heat has sunk a few degrees below the standard."

We are induced to insert the Table which follows, bearing out, as it fully does, the preceding observations.

The Temperature and Mean Weekly Deaths in the Metropolis, for periods of Four Weeks, from November 7th, 1840, to April 17th, 1841.

| | November 7th—28th. | November 29th to December 26th. | December 27th to January 23rd. | January 24th to February 20th. | February 21st to March 20th. | March 21st to April 17th. |
|---|---|---|---|---|---|---|
| | ° | ° | ° | ° | ° | ° |
| Temperature { Mean | 46·3 | 35·8 | 34·3 | 37·0 | 46·0 | 48·5 |
| Temperature { Lowest | 33·0 | 21·0 | 15·0 | 22·0 | 34·0 | 37·0 |
| Weekly Deaths ...... | 905 | 1,086 | 1,239 | 1,056 | 1,039 | 844 |
| Age, 0—15 ........ | 438 | 512 | 513 | 451 | 443 | 366 |
| ,, 15—60 ........ | 292 | 336 | 393 | 355 | 341 | 292 |
| ,, 60 and upwards.. | 171 | 235 | 336 | 250 | 253 | 185 |
| From Bronchitis ...... | 6 | 18 | 34 | 24 | 19 | 15 |
| ,, Pneumonia .... | 100 | 138 | 140 | 97 | 89 | 62 |
| ,, Asthma ........ | 20 | 55 | 92 | 56 | 43 | 23 |

Mr. Farr adds that, so far as statistical investigation has hitherto gone, temperature appears to have no influence on the fatality of consumption (tubercular phthisis); while it exercises a well-defined influence in emphysema and in the inflammatory diseases of the chest.

We cannot conclude this account of Mr. Farr's letter without expressing our high sense of the industry and acumen of its author. He deserves in an eminent degree the thanks of the profession and the public, and we are satisfied that his analyses of the mortuary tables of this country are calculated to prove beneficial to science, and to re-act beneficially on the health of the community. They dispel some professional as well as popular errors, and establish some far from unimportant truths.

